# Consumers’ Attitude towards the Sustainability of Different Food Categories

**DOI:** 10.3390/foods9111608

**Published:** 2020-11-05

**Authors:** Paola Sánchez-Bravo, Edgar Chambers, Luis Noguera-Artiaga, David López-Lluch, Edgar Chambers, Ángel A. Carbonell-Barrachina, Esther Sendra

**Affiliations:** 1 Research Group “Food Quality and Safety (CSA)”, Department of Agro-Food Technology, Escuela Politécnica Superior de Orihuela (EPSO), Universidad Miguel Hernández de Elche (UMH), Ctra. de Beniel, km 3.2, 03312 Orihuela, Spain; paola.sanchezb@umh.es (P.S.-B.); lnoguera@umh.es (L.N.-A.); angel.carbonell@umh.es (Á.A.C.-B.); 2 Center for Sensory Analysis and Consumer Behavior, Kansas State University, Manhattan, KS 66502, USA; echambersv@gmail.com (E.C.V); eciv@ksu.edu (E.C.IV); 3 Department of Agro-Environmental Economics, Escuela Politécnica Superior de Orihuela (EPSO), Universidad Miguel Hernández de Elche (UMH), Ctra. de Beniel, km 3.2, 03312 Orihuela, Spain; david.lopez@umh.es

**Keywords:** consumers’ perception, education level, environmental issues, generations, income

## Abstract

Currently, poverty, climate change, environmental pollution and the depletion of natural resources have generated a greater concern for sustainability. The objective is the survival of the human species and the persistence of all components of the biosphere. To achieve sustainability, human participation is essential; sustainable consumption depends on consumers’ perceptions of sustainability and how they affect their behavior. The aim of this study was to understand consumers’ perceptions and attitudes towards food sustainability based on country, age, gender, income and education level. An online survey was carried out in countries in Europe, America and Asia. Consumers were asked questions organized into food categories. The results showed that consumers’ attitude towards sustainability is understood differently in each country, even within the same food category. Consumers with lower education level showed the lowest knowledge and concern about food sustainability. Older generations were less aware of sustainability and its related problems. While income level presented unclear results, gender did not affect attitude towards food sustainability. Therefore, to achieve a sustainable future, raising awareness among the population is increasingly necessary. Consequently, segmenting training campaigns according to the group they are aimed at will provide a greater impact and, therefore, greater awareness.

## 1. Introduction

Sustainability is more and more important due to current world problems such as poverty, climate change, environmental pollution and the finiteness of natural resources [1]. Sustainability arose as a concept in 1987, when it was defined in the Brundtland Report in terms of a goal: to “meet the needs of the present generation without compromising the ability of future generations to meet their own needs” [2]. Since then, many ways of defining sustainability have come emerged. Brown, et al. [3] pointed out some common features that are involved in the concept of sustainability: (i) the continued support of human life on earth; (ii) long-term maintenance of the stock of biological resources and the productivity of agricultural systems; (iii) stable human populations; (iv) limited growth economies; (v) an emphasis on small-scale and self-reliance; and (vi) continued quality of the environment and ecosystems.

The key aspect in defining sustainability is its anthropocentric perspective. The real objective is the survival of the human species across all regions of the world and the persistence of all components of the biosphere, even those with no apparent benefit to humanity [3]. However, all these depend on human behavior and supposes, at least, three core dimensions of sustainability [4]: (i) environmental (protection of natural environment and resources), (ii) social and cultural systems, and (iii) economics (promotion of decent human living conditions).

These three aspects are linked to consumption consequences [5]. In this sense, sustainable consumption and production are defined according to the United Nations Environment Programme (UNEP) [6] as a “holistic approach to minimize the negative environmental impacts from consumption and production systems while promoting quality of life for all”. Paavola [7] provided the definition of sustainable consumption as “consumption that entails a reduction of the adverse impact on the environment”.

Food consumption, distribution and production are key aspects of human life. There are several studies of the relation between food consumption and sustainability [8,9,10]. It is clear that consumers have a major role in making food chains more sustainable through the choices they make when buying food because these give information to producers about which foods, how they should be produced and where they must be sold [11]. In this sense, it could be said that, although the way food is produced can be changed, market forces (consumer demand) are one of the most important factors in the development of food chains [12].

Sustainable consumption patterns depend on the perceptions of sustainability that consumers have, how these perceptions build attitudes and how these attitudes affect their behavior. Consumer perception can be understood as the translation of sensory perception into buying behavior. In this sense, sensory perception is linked to the way that humans perceive and process sensory stimuli, but not only through their senses. Consumer perception relates also to how opinions about companies and their products are made. In the other direction, companies use consumer perception theory, firstly, to understand what consumers think about them and their offers and, secondly, to develop communication strategies to build loyalty of current consumers and to attract new ones.

As sustainability is linked to environment, it can be said that perception about sustainability is related to environmental concern. This concept can be defined as “an individual’s perception and conviction that humans endanger the natural environment combined with the willingness to protect it” [13]. Researchers use this term to refer to the whole range of environmentally related perceptions, emotions, knowledge, attitudes, values and behaviors [14]. Thus, environmental concern would link perceptions, attitudes and behaviors. This concept holds, according to Franzen and Vogl [15], at least, three aspects: (i) the rational awareness of the problem, (ii) the emotional affection caused by the problem and (iii) the willingness to act to solve the problem.

Environmental concern can be analyzed as an awareness of consequences, using Schwartz’s [16] norm activation theory of altruism. Empirical evidence shows that environmental concern has major effects on pro-environmental behavior and hence on sustainability perceptions and attitudes. In this sense, it would induce a sense of responsibility, a commitment to behave by following personal rules or moral obligations leading to environmentally protective actions [17]. Pro-environmental norms reflect the extent to which a person feels a personal obligation to contribute to the solution of an environmental problem [18].

Attitudes about environmental issues depend on the relative importance that a person places on himself/herself, humankind and the whole planet [19]. According to Stern and Dietz [20], these attitudes can be linked to environmental consequences, labelled as egoistic, social-altruistic, and bio-spheric consequences and related to three different underlying value orientations. So it can be said that environmental concerns arise because people become aware of harmful consequences to something that they value. However, this awareness is going to depend on people’s perceptions. These perceptions will build people’s attitudes and values.

An important aspect that derives from values is trust. Trust generally is understood as a situation characterized by the following aspects: one party (trustor) is willing to rely on the actions of another party (trustee); the situation is directed to the future. The trustee has control either because the trustor gave up that control or, in the case of environmental or sustainability issues, because the trustor has no way of confirming the action. This is included in the introduction as it is a key part of how values are created in a society. In this sense, environmental concern and perceptions on sustainability perception depend on values and on the degree of trust. It can be assumed that trust is linked to a strong concern about environmental issues. People show different levels of confidence towards other people and institutions. Trusting other people increases environmental concern [21] as it creates the belief that others are also concerned about environmental issues and are helping to provide and maintain public goods. According to this, trusting public institutions should also influence people’s environmental concern. However, this is not so clear. Two questions have to be addressed. First, public institutions are responsible for providing public goods. People that do not trust public institutions can tend to think that environmental problems are not properly considered. People could be less ready also to provide public goods or services when they think that others (public institutions) are not fulfilling their tasks. So this is not a resolved aspect of the problem and, most importantly, it depends on people’s perceptions about sustainability.

It is interesting to look at personal and national differences regarding environmental concern and perceptions and attitudes about sustainability. In this sense, environmental concern is strongly related to national wealth. People living in wealthier countries show higher environmental concern. The wealth of a country has a positive effect on individual environmental concern [19].

However, individual differences within a country are more defined than differences among countries. According to Franzen and Vogl [15], persons’ environmental concern relies on socio-demographic characteristics such as gender, age, income and education. This could be explained looking at different social roles. Younger people show higher concern than older ones as they have grown up in times when the media focused more attention on the problem. However, environmental concern increases first and then drops as people get older. Income level is also related to environmental concern. The higher the income, the higher the concern about environmental problems. This can be explained looking at two facts. First, richer people do not worry about personal economic problems, so they can look at other questions. Second, richer people normally maintain a higher consumption of private goods and a higher demand for public goods. They present a higher willingness to pay for better goods. Finally, education is directly related to environmental concern [22]. The higher the knowledge about environmental problems, the higher the concern.

Furthermore, value orientations are also related to environmental concern. This can be understood looking at Inglehart’s post-materialism hypothesis [23]. This theory proposes that societies face changes as they develop economically. Economic change creates generations with higher materialistic values (e.g., the desire for economic growth and price stability). Generations that grow in economic prosperity show stronger post-material values (freedom and self-realization). Post-material values are positively linked to environmental concern as economic prosperity is no longer a question to be solved.

Environmental concern becomes environmental behavior when people decide to act. A group of environmental behaviors are linked to the idea of frugality (use reduction, recycling and re-use of objects) [24]. A frugal attitude is linked to cooperative behavior in resource dilemmas, or to resource conservation behavior [25]. It requires motivation to save resources, an idea of “efficiency” and a strong confidence in other people. Obviously, these motivations depend on perceptions and attitudes about sustainability.

Consequently, sustainable food consumption has to be understood as a behavior that depends on perceptions about what a consumer buys and how production and distribution affect the environment. These perceptions can lead to attitudes that then predict behavior. This is why looking at people’s perceptions about sustainability is a key factor to understand how and why they buy and eat food products.

The fact is that the more local and the more seasonal a food product is, the more sustainable it usually is, but this idea is not understood by the majority of population. As has been said, dietary change can deliver environmental benefits on a scale not achievable by producers [11]. Consumers can play another important role by avoiding high-impact producers [12]. This action needs previous awareness, and awareness depends on perceptions and attitudes. So communicating average product impacts to consumers is the first step to making dietary change possible, as it can help to change perceptions and, hence, attitudes. However, producers and society need to know which are these perceptions and attitudes and which are the main groups with faulty ideas about sustainability in order to develop communication strategies. The first hypothesis of this research was that consumers do not clearly understand the concept of environmental sustainability regarding food. The second was that consumers’ perception of this concept differs depending on cultural background and personal characteristics. Thus, the main objective of this study was to understand consumer’s perceptions and attitudes regarding sustainability in different food categories.

## 2. Materials and Methods

The development of a scale for measuring thoughts about sustainability is important to understand people’s perceptions on sustainability, and how producers can attract consumers willing to buy goods that are eco-friendly and/or who want to support small producers.

A questionnaire was developed for a global study on sustainability, which was conducted with more than 3600 participants in 6 countries (Brazil, China, India, Mexico, Spain and the United States). The survey was completed by 50% of self-identified men and women. Four age ranges were selected (25% of participants for each age range), clearly differentiated: 18–23 years (centennials); 24–41 years (millennials); 42–52 years (gen X) and 53–73 years (baby boomers). Five levels of study were evaluated (primary school or less, high school diploma, associate’s degree, bachelor’s degree and graduate degree or higher) and 4 income ranges (25,000 US dollars or less, 25,001–50,000 US dollars, 50,001–100,000 US dollars and more than 100,000 US dollars). For income, each country studied adapted ranges (in the official currency of each country) to obtain results from the lower, middle and upper income classes of each country. Results for income have been expressed in US dollars.

The present study was conducted by Qualtrics (an online survey company). The survey was launched simultaneously in the six countries studied. Respondents did not receive a financial incentive; however, the Qualtrics database has a reward system to compensate respondents for their time and collaboration. The questions selected for the study were established through an expert discussion and following the model used by Sánchez-Bravo et al. [26].

The questionnaire used in the current study was included in a large survey analyzing multiple aspects of the sustainability of food categories. The questions were organized into 13 food categories: (I) bread and cereal products; (II) snacks; (III) sugar and derivatives; (IV) fruits and vegetables; (V) fats and oils; (VI) coffee, tea and cocoa; (VII) soft drinks and water; (VIII) alcoholic beverages; (IX) meat products; (X) eggs; (XI) milk and dairy products; (XII) fish and seafood; and (XIII) food for special dietary uses.

The questions were presented within the questionnaire in a random order. Socio-demographic questions were also evaluated. The survey was translated into five languages (English, Spanish, Portuguese, Hindi, and Mandarin Chinese). Verification of the translations was performed through a back translation. The survey was conducted online and was presented in each country in its most common official language. A cheating question was introduced to avoid consumers from responding randomly, (“salt is a flavor enhancer and I am trying to double my intake of salt”). Questionnaires containing the wrong answer for such question were removed from the study.

Responses were measured on a Likert type scales of 7 points (1: strongly disagree, 2: disagree, 3: disagree somewhat, 4: neither agree nor disagree, 5: agree somewhat, 6: agree, 7 strongly agree). Demographic data were obtained by multiple choice answers. The full questionnaire is presented in Table 1.

### Statistical Analysis

The results were processed by one-way analysis of variance (ANOVA) followed by Tukey’s multiple range test, with a confidence interval of 95% and significant difference was defined as *p* < 0.05. Reliability was tested using Cronbach’s alpha (95% confidence), while questions were clustered using principle component analysis (PCA), both of which were ran using the software R (programming language). We also used PCA to cluster the questions and avoid similar questions. Euclidean distance by an agglomerative hierarchical method (Ward’s) was used to group consumers into clusters. Software XLSTAT (2016.02.27444 version, Addinsoft, Paris, France) was used.

## 3. Results and Discussion

The total number of questions selected for analyzing the data was reduced to 19 (highlighted in grey color in Table 1) through Cronbach’s alpha analysis and PCA analysis). These questions were the least similar to each other and, therefore, the most representative. Results are shown in Table 2, Table 3 and Table 4 and are divided into different food categories for easier discussion and understanding.

**Bread and cereals**. Regarding grains, the most representative questions were related to water consumption. Consumers from all countries, ages, genders and income level agreed on the need to select proper grain varieties with low water requirements to decrease their environmental impact (Table 2, Table 3 and Table 4, Q2.29). Only education level (Table 4, Q2.29) significantly affected this perception, consumers with the lowest educational level being those that considered such selection of grains less necessary. Regarding the willingness to consume an ancient grain, sorghum, which has low water requirements (Table 2, Q2.30) as a substitute for corn/wheat, there were significant differences due to country, age and educational level. The most developed countries (USA and Spain) felt reticent to replace wheat or corn, even if products made with sorghum tasted good. The fact that the USA is the largest corn producer, with almost 400 million tons, and wheat is the third most produced commodity in Spain, behind olives and barley [27], may have contributed to this reticence to replace corn and wheat. Worldwide consumers older than 42 and those consumers with only primary education were also reticent (Table 2, Table 3 and Table 4, Q2.30). This behavior was expected because environmental concern decreases as people’s age increases [15,22,26]. However, interest in heritage cereals is increasing even among older consumers, with farmers and consumers tending towards local and sustainable production and purchase. In this sense, Wendin, et al. [28] showed that, although all consumers know about heritage cereals, their consumption is affected by geographical area, but not by level of education. Added to this is the concern of older consumers for their health and their willingness to pay more for traditional cereals.

**Sugar and derivatives**. Factors affecting perception of sugar sustainability were country and education level (Table 2, Table 3 and Table 4). Sugar consumption is very high worldwide. Every year, an average of 24 kg per person of sugar is consumed. However, in developed countries (European Union, USA, Canada, Australia and New Zealand) this consumption increases up to 35.5 kg [29]. Consumption is even higher in Mexico and Brazil (38.7 and 67.3 kg, respectively), the latter being the country that consumes the most sugar in the world [29]. On the other hand, China and India have a lower sugar consumption than the world average (11.7 and 20 kg, respectively). In this sense, Mexico and Brazil were the countries that were more willing to reduce their consumption if it helps food chain sustainability (Q2.17; 5.3 and 5.1, respectively). On the other hand, US consumers disagreed the most (4.3), as was the case worldwide with consumers of lower educational level (4.6). In the USA, sugar consumption, especially in sugar beverages, is widely spread [30] and its high consumption is linked with obesity, cardiovascular disease and diabetes [31]. Therefore, the World Health Organization (WHO) made a recommendation to consume less than 10% of necessary calories in the form of sugar in 2014 [32]. Furthermore, nutritional warnings, especially negative ones about health, are an important factor for consumers when buying a product [33]. In 2019, China, India and USA were the countries with the highest number of diabetes patients, although diabetes prevalence is relatively low (10.9, 8.9 and 13.3%, respectively) [34]. On the other hand, the diabetes prevalence of Brazil and Mexico reached 11.4 and 15.2% respectively [34]. In addition, Mexico was the country with the highest percentage of population with obesity or overweight, exceeding 75% [35]. Reducing sugar consumption is one of the main strategies to reduce obesity and overweight [36]. In this sense, due to the high incidence of diabetes among the population of Mexico and Brazil caused by high sugar consumption, and the high rate of overweight reported, it is possible that reducing sugar consumption is the strategy to be followed by these countries to reduce diabetes and diseases derived from the consumption of sugar, along with the overweight and obesity of the population.

**Fruits and vegetables**. Taking a view on seasonal fruit consumption (Table 2, Table 3 and Table 4, Q2.34), gender and income did not affect consumer preference. On the other hand, country, age and educational level significantly affected perceptions regarding sustainability in seasonal fruit and vegetables consumption. It is widely known that seasonal fruit consumption is more sustainable than that of non-seasonal fruits [37]. However, consumers in the USA, Brazil and China (5.1, 5.1 and 5.2, respectively) were not aware of issues of sustainability in seasonal fruit consumption. Worldwide consumers of the highest and lowest age ranges and consumers who only had primary school education did not consider seasonal fruits as more sustainable. This is because, in general, a higher level of education implies a greater concern for environmental problems and sustainability [38], and possibly knowledge of fruit seasonality. On the other hand, seasonal fruits and vegetable consumption is based on flavor and freshness, whereas reducing environmental impact is seen as a secondary factor [37].

When asking about fresh versus dried fruits (Table 2, Table 3 and Table 4, Q2.26), only country and income factors affected responses. In India, fresh fruit consumption was not considered more sustainable than dry fruit consumption (4.5). This is consistent with the fact that fresh and dry fruit productions are an important part of the Indian economy [39]. On the other hand, consumers with the highest level of income were more aware of this fact (5.2). In this sense, Brooks et al. [37] found that a major part of food environmental impact comes from processing. Therefore, a processed product is going to be less sustainable than its fresh equivalent. Furthermore, Sánchez-Bravo et al. [26] demonstrated that consumers associate highly processed products, such as snacks, with a lower perception of “sustainability”. On the other hand, the reasons for choosing a more or less processed food depend on lifestyle. The most active consumers prefer to buy less processed foods [40].

Regarding the use of traditional varieties to avoid loss of biodiversity (Table 2, Table 3 and Table 4, Q2.41), country and level of education were the factors that showed significant differences, in contrast to age, gender and income. Currently, loss of biodiversity is one of the biggest problems; it is not only an environmental problem, but also basic in ensuring food security. Consumption patterns and intensification of agriculture are the main causes of biodiversity loss. Promoting the use of traditional and indigenous varieties is one of the main mechanisms of action to avoid and/or manage biodiversity losses [41]. In this sense, consumers from India, Mexico and China gave the greatest importance to traditional varieties as an alternative to avoid the loss of biodiversity (4.8 and 4.9, respectively). The contrary happened with consumers worldwide with the lowest education level. It is widely accepted that a greater crop diversity and, therefore, smaller and/or adapted agricultural production positively influence the development of more sustainable and economically stronger agro-food systems [42,43]. Likewise, increasing biodiversity requires consumer acceptance of diverse products [42]; thus, it was not surprising that developing countries, such as India, Mexico or China, were more open to the consumption of traditional varieties. Furthermore, it was also to be expected that less academically educated consumers would be less aware of the problems arising from the loss of biodiversity (Table 4).

**Fats and oils**. Looking at olive oil as more sustainable than canola oil (Table 2, Table 3 and Table 4, Q2.11), gender, income and education level were the factors that did not affect consumer perception. By contrast, country and age showed significant differences. Olive oil is widely consumed in Spain [44,45], therefore it was expected that consumers in this country would have considered it more sustainable than canola oil. On the contrary, USA and China consume much less olive oil than soybean and canola oil [46], so it was not surprising that they were the countries that least agreed (4.4in both countries). Besides, younger generations worldwide did not consider olive oil as more sustainable than canola oil (Table 3). These results contrast with those obtained by Bollani, et al. [47], who established that, in general, the Millennial generation are more concerned with sustainability and environmental issues.

Regarding reducing animal fats as a sustainability enhancer (Table 2, Table 3 and Table 4, Q2.32), country, age and education level were the factors that affected perceptions. China, Mexico and Brazil agreed with the fact that reducing the consumption of animal fats is a sustainable behavior, while the USA and Spain were less in agreement, as well as younger and less educated consumers worldwide. Animal fats (tallow, butter, lard, etc.) are widely consumed today. The USA produces a large amount of tallow and fats, so it makes sense that it did not seek to reduce the consumption of this type of fat [46]. In Spain there are many traditional processed cured meat products containing high amounts of animal fat. Originally such meat products allowed the preservation of meat and the reduction/avoidance of food waste. On the other hand, it seems that younger generations worldwide are still not aware of the importance of reducing animal fat consumption, for both sustainability and health.

**Coffee, tea and cocoa**. With regard to the category “coffee, tea and cocoa”, factors that showed significant differences were country, age and education level. Mexico, Spain and India were the countries that most agreed (4.8, 4.9 and 4.9, respectively) that a higher price should be paid for coffee beans from small farms, because this is fair for farmers (Table 2, Table 3 and Table 4, Q2.4). On the other hand, the USA and Brazil were less in agreement with this statement (4.4 both countries). Sánchez-Bravo et al. [26] showed that US consumers do not consider small farmers as essential in maintaining sustainability. Furthermore, Brazil is the main coffee producer [27], so it was to be expected that they did not want small producers to be encouraged. This behavior was repeated in worldwide consumers over 42 years old and in consumers with an associate’s degree or less (Table 3 and Table 4). This showed that education is key when it comes to buying sustainable products: “the greater knowledge, the greater concern” [48].

Taking a view on fair trade certification (Table 2, Table 3 and Table 4, Q2.18), Brazil and India considered that fair trade certification guarantees the origin of the product and, therefore, makes the consumer feel better (5.0 and 4.9, respectively). Consumers between 24 and 41 years old worldwide, as well as those with the highest education levels, showed similar behavior (Table 3 and Table 4). In contrast, USA and Spain did not value fair trade certification as much (4.5 both countries). The certification process of a product is key in establishing its acceptance among consumers [26,49]. In India, the consumption of organic food suffered in obtaining acceptance due to the lack of official certification that guarantees its origin [26,50]. On the other hand, in general, consumers worldwide have favorable expectations of foods labeled as “organic,” and of other labels such as “eco-friendly,” “local,” “fair trade” or “natural”. This effect is known as the “halo effect” [42]. In fact, research has shown that use of the term “organic” implies “naturalness” to consumers who may think those ingredients and products are more sustainable too [51,52]. However, consumers with health problems are less affected by this effect [42,53]. Older people have more health problems [54]. This seems to indicate why older consumers were less in agreement with fair trade certification. Also, younger generations worldwide show higher concern about environmental issues [26,47].

**Soft drinks and water**. Regarding returnable soft drinks consumption in glass bottles (Table 2, Table 3 and Table 4, Q2.21) country, age and income were important factors with respect to this category. China, Mexico and Spain were more in favor of consuming soft drinks from reusable glass bottles (5.6, 5.6 and 5.8, respectively). The use of returnable glass bottles reduces the eutrophication of the soil and the waste generated [55]. However, the use of returnable bottles requires inspection and decontamination of returned bottles [56]. This implies costs that, according to results, not all countries are willing to assume. Furthermore, sustainability of returnable glass bottles depends on the number of reuse cycles [55]. In contrast, oldest and least educated consumers worldwide gave the lowest scores (5.1 and 4.9, respectively) in willingness to buy returnable glass bottles. This reinforces the previous statement that education and age are determining factors in sustainable consumer behavior.

**Alcoholic beverages**. When asked about home distilled alcohol consumption (Table 2, Table 3 and Table 4, Q2.15), country and income factors significantly affected response. Alcohol consumption is related to an increased risk of cancer, stroke and liver cirrhosis, in addition to the social consequences derived [57]. Consumers from the most developed countries (Spain and USA) are those with the highest alcohol consumption (10.4 and 8.9 L per capita, respectively) [57]. Consumers in the USA and China were the least concerned about the risk of consuming home distilled alcohol (4.4 both). In the same way, consumers worldwide whose income level exceeds 25,001 US dollars agreed the least with the consumption of home distilled alcohol. Income level is also related to environmental concern in some cases [26]. The higher the income, the greater the concern for sustainability. This is reinforced by our results (Table 4).

**Meat products**. Regarding the question that providing a low environmental impact certification would increase meat consumption (Table 2, Table 3 and Table 4, Q2.2), country, age and education level showed significant differences in response. In general, Mexico and Brazil were the countries that indicated a more favorable opinion (4.8 and 4.7, respectively), as well as consumers worldwide under the age of 52 and with high school and primary school education or less. In contrast, US and Indian consumers disagreed that certification would help to increase their meat consumption. As previously mentioned, the certification of a product is an important factor in the consumer purchase decision [26,49]. In this sense, trust in certification agencies is key. In the USA, consumers have full confidence in their certification agency (USDA: United States Department of Agriculture), while in India the certification system is less reliable [49]. This fact seems to explain why certification will not help Indian consumers to eat more meat. Furthermore, India is the main country with vegetarian populations, having the lowest meat supply in the world (3.78 kg per person) and most of the beef cattle is destined for dairy production [27,58].

Looking at beef cattle sustainability (Table 2, Table 3 and Table 4, Q2.6), only country and income factors presented significant differences. Mexico, Brazil and India considered that beef cattle is not sustainable due to the high carbon footprint it generates (Table 2). This behavior was also shown among consumers worldwide with the highest level of income. Currently, meat products represent 30% of total world food consumption [59]. Livestock production causes an increase in greenhouse emissions and negatively influences the water footprint and water pollution [60]. Therefore, reducing this type of meat would help to lower greenhouse gases and their impact on climate change. In addition, avoiding red meat consumption would help in the prevention of diseases related to the consumption of this type of meat: cardiovascular diseases and some types of cancer such as colorectal cancer [60]. Therefore, providing information to the consumer is essential in guaranteeing a sustainable future. Since livestock is a source of greenhouse gas emissions, the “low carbon diet” has become a new trend. Currently, to partly replace meat, meat diluents and other non-meat substances (with a high protein content) are being used. These products offer opportunities for reformulation of more healthful and sustainable meat products [61]. However, although environmental concern affects purchase intention, it is still vegetarian consumers who are willing to pay more for this type of product [62].

**Eggs**. Taking a view on the eggs category, factors that showed significant differences were country and age. Spain and Brazil think that eggs from free-range hens are more sustainable than those from caged-hens (Table 2, Table 3 and Table 4, Q2.8; 5.6 and 5.4, respectively) and, together with Mexico, they would prefer to be able to buy them directly from the farmer (Table 2, Table 3 and Table 4, Q2.23). In contrast, US and India consumers were the least in agreement with these claims. These results are related to those obtained by Rahmani, et al. [63] who found that free range chicken eggs were the preferred option for Spanish consumers. Consumers worldwide of the oldest generation thought that free-range chicken eggs are not more sustainable and were less willing to buy eggs directly from the farmer (5.9 and 5.5, respectively). In general, older people tend to have mobility problems, so traveling to the farm would be an extra effort that many of them could not perform. This fact could explain why consumers older than 53 years were reluctant to buy eggs directly from the farmer.

**Milk and dairy products**. Regarding milk traceability as an important aspect in the decision to purchase dairy foods (Table 2, Table 3 and Table 4, Q2.40), country and income were the factors that presented significant differences. In Brazil and China, consumers considered milk traceability key when buying cheese (4.9 and 5.0, respectively). This aspect was also important for consumers with an income level above 100,000 US dollars. Milk production intensification in Brazil in the last decade has increased. This has provoked environmental and economic stress [64]. In this sense, Brazilian consumer’s concern about milk traceability could be a way of ensuring its local origin and, thereby, reducing environmental impact caused by increased production.

Looking at home-made yogurt consumption as more sustainable than that of yogurt bought from the store (Table 2, Table 3 and Table 4, Q2.38), only gender did not affect response. Country, age, income and education level were the factors that showed differences. Brazil and India were in agreement with home-made yogurt consumption (5.4 and 5.3, respectively). In contrast, in the USA consumers opted for the consumption of yogurt made away from home (4.1). This opinion was shared by older consumers, consumers with incomes above 25,001 US dollars and consumers with an education level between high school and bachelor’s degree.

On the other hand, country, age and education level were the factors that affected the question relating to milk consumption as being more sustainable than that of its derivatives (Table 2, Table 3 and Table 4, Q2.25). China and India consumers (4.7 and 4.8, respectively), younger generations and, in general, higher education levels, considered eating milk more sustainable than eating cheese. In general, the wealth of a country has a positive effect on environmental concern [19]. Therefore, it was to be expected that the USA and China would be the most aware. However, the USA did not behave as expected. On the other hand, results obtained for the factors of age and education strengthen the previously established fact that young generations and a high level of education lead to a greater concern for sustainability and the environment (Table 3 and Table 4).

In summary, the determining factors in attitude of consumers to sustainability were country, age and education (Table 5). As mentioned above, sustainability depends on human behavior [3]. Today, many studies have been carried out on the sustainability of water in agriculture products, called “hydroSOS” ([65,66,67,68,69,70,71] and, in addition, hydroSOStainability markers have been determined, through which certification protocols have been prepared, both in the field and for the product itself [72,73]. In this way, it is produced in a more sustainable way and, in addition, the farmer obtains an additional benefit, not only in saving on the cost of water, but also in obtaining higher quality fruits [65,66,70,71,74], generating a cycle that culminates with the purchase of the product by the consumer. However, if the consumer is not able to identify or value sustainable products, the cycle is broken. Therefore, to achieve a sustainable future, raising awareness among the population is increasingly necessary. Consequently, segmenting training campaigns according to the group they are aimed at will provide a greater impact and, therefore, greater awareness.

Recent studies indicate that consumers interested in organic or local products could be the key to sustainable consumption. This consumer profile might accept new forms of production and new foods, such as those made with recycled ingredients [75].

### Consumer Clustering

**Figure 1:**
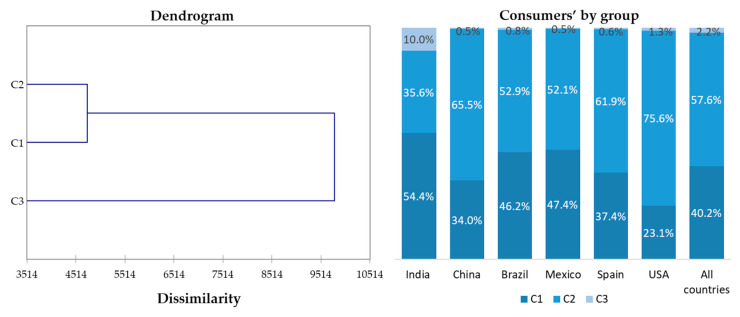
Dendrogram showing Euclidean distances between clusters and percentage of consumers of each group.

Clustering (Figure 1) was carried out to group the different consumers studied according to their interest/knowledge of sustainability. Three main groups (C1, C2 and C3) were found with C1, consisting of 40.2% consumers (those highly interested in sustainability), C2 including 57.6% consumers (those consumers with some interest and concern about sustainability, but not at the highest level), and C3 representing consumers who were not at all concerned about sustainability. C3 included only 2.2% of the overall population of consumers in the countries studied.

Consumers in C1 scored the highest for the questions most related to sustainability, while those in C2 scored somewhat lower, and consumers in C3 scored far lower on average than all other consumers for those questions (Table 6). For example, the mean scores for the 19 selected questions (Table 3 and Table 4) ranged from 4.67 to 6.05 for C1, those most interested and concerned about sustainability. The mean consumer scores for C2 were lower than C1 by 0.5 to 1.5 points for all questions and ranged from 3.87 to 5.31. Scores for C3 were quite low (means = 1.42 to 2.12) indicating little or no interest in or concern about sustainability. Other authors have shown varying percentages of interest in specific products that have some sustainability attributes. For example, about 25% of consumers were not interested in sustainability labels for chocolate [76]. However, Dagevos and Voordouw [77] noted that percentages of consumers who were “willing to pay” for sustainability vary around the world and more consumers’ indicate an interest in sustainability than actually show sustainable behaviors.

In this research, consumers in the USA were in the C2 group (75.6%) to a greater extent than in other countries. That is, they were concerned to a medium degree about sustainability. This was similar to consumers in China and Spain, although the percentages were slightly lower in that category. On the other hand, Indian consumers showed contradictory behavior. More than half of Indian respondents (54.4%) were highly concerned about sustainability (C1), but, at the same time, India was the country with the highest percentage of consumers included in group C3 (10%), those who were not concerned with sustainability. Consumers from Brazil and Mexico were more equally divided between groups C1 and C2.

As mentioned previously, consumers in rich countries are more likely to show greater environmental concern. People who grow up under a prosperous income level, which guarantees their economic well-being, are generally more concerned about environmental problems [19]. This is reflected in the behavior of consumers in India, where the level of wealth is high in a portion of the population, but a significant percentage of the population is poor.

## 4. Conclusions

In general, it seems that consumers have not yet internalized environmental sustainability. Likewise, sustainability is understood differently in the different countries studied and depends on food category. Consumers of low educational level and older generations are less aware of and less interested in sustainability and the problems derived from it. Income level, while key in some food categories, is not fully indicative of consumer awareness of sustainability. Gender does not affect this same awareness, based on this data. Consumer awareness is key to achieving sustainability. In general, it can be seen how the level of education and age are the main factors that account for differences in consumers’ concern related to food sustainability.

Because sustainability is understood differently in the countries studied and depends on food category and consumer demographics, it is crucial that policy makers develop a strategy for providing meaningful, accurate information about sustainability to various groups. How sustainability affects the specific foods people eat, the production processes for those foods, and how consumer behavior affects sustainability is one of the most important drivers for increased interest in sustainability. Because educational level and age were also found to be determinants for this perception, actions should be focused on promoting awareness among groups that show higher misperception. Starting by targeting information campaigns towards consumers with a lower level of education, segmented for each age and country, will be essential.

## Figures and Tables

**Table 1:** Full questionnaire.

Number	Question
Q1	Demographics
Q2	Please indicate your agreement to the following questions.
Q2.1	A nice plastic packaging is essential to sell sustainable snacks.
Q2.2	A certification proving low environmental impact will help me in eating more meat products.
Q2.3	Grains are always grown under rain-fed conditions, not with irrigation.
Q2.4	Coffee beans from extremely small farms in the mountains of Jamaica deserve a high price to be fair to the farmers and support their economic future.
Q2.5	Consuming farm raised fish is less sustainable than consuming sea-fish.
Q2.6	Beef cattle are not sustainable because they have a high carbon footprint.
Q2.7	Eating seafood can be risky due to the occurrence of mercury but it is so delicious that I cannot stop eating it.
Q2.8	Eggs from free-range hens are more sustainable than those from caged-hens.
Q2.9	Even though corn requires a high volume of water, I will never stop buying corn products because I like them too much.
Q2.10	Even though I know that confections and cakes may not be good for my health, I cannot help it and still consume them often.
Q2.11	Extra virgin olive oil is more sustainable than canola oil because it does not go through a refining process.
Q2.12	Product labeled “Fair Trade” (e.g., some coffees or chocolates) are too expensive.
Q2.13	For sure I will buy brown sugar if it is labeled with a high carbon and water footprint.
Q2.14	Green-house vegetables are widely available and are safer and more sustainable than the rain-fed ones.
Q2.15	Home distilled alcohols cannot be considered sustainable if they risk the consumers’ health in any way.
Q2.16	I do not think milk quality varies much and will buy any that is on sale.
Q2.17	I will reduce my consumption of discretionary products (oils, sugars, alcohol) if that helps in the sustainability of the food chain.
Q2.18	I like to buy fair trade products, because this certification makes me feel good about the product source.
Q2.19	It is important to eat a variety of vegetables even if they are out of season.
Q2.20	I will not buy bottled water because it is less sustainable than tap water.
Q2.21	I would buy soft drinks in glass bottles if they were returnable and I will be rewarded.
Q2.22	If I had known that a particular food (for instance, oranges) is one of the foods producing more environmental impact, I would have reduced its consumption before.
Q2.23	If I could, I would prefer to buy eggs directly from the farmer.
Q2.24	Independent of the taste, drinking bottled water is safer than drinking tap water.
Q2.25	It is more sustainable to drink milk than eat cheese.
Q2.26	It is more sustainable to eat fresh fruit than dry fruit because energy is needed to dry the products.
Q2.27	Organic vegetables are the perfect choice for consumers because they are bacteria-free.
Q2.28	Packing cupcakes individually in crystal clear bags within a bigger plastic bag is sustainable because it allows consumption of smaller portions.
Q2.29	Proper selection of grains with low water requirements will help to protect the environment.
Q2.30	If I knew that an ancient grain, such as sorghum, uses less water than wheat or corn I would want products made from that if they tasted good.
Q2.31	Providing consumers that have special dietary needs with proper foods is also part of the sustainability of the food chain.
Q2.32	Reducing the intake of animal fats can be considered as a sustainable behavior because it reduces medical expenses.
Q2.33	Rice-based food for people with gluten sensitivity must be sustainable to ensure the best possible quality and safety.
Q2.34	Seasonal fruits are the most sustainable foods; they can be eaten directly from the plant.
Q2.35	Sustainable snacks are those prepared using grains that optimize the use of irrigation water.
Q2.36	The high demand for palm oil is seriously jeopardizing the forest in countries such as Indonesia and Malaysia.
Q2.37	The information on labels of snack food is so much that I just buy snacks from the best-known brands.
Q2.38	Making yogurt at home is more sustainable than buying it from the store.
Q2.39	Canned-fish products are non-sustainable because they generate tons of waste.
Q2.40	The traceability of the milk (where the milk comes from) used in the cheese I eat is an important buying driver for me.
Q2.41	Traditional peach varieties are sustainable because they increase biodiversity.
Q2.42	Vodka is a sustainable drink because is prepared using cereal grains or potatoes.
Q2.43	When drinking alcoholic beverages, I do not care about their nutrition or health effects.
Q2.44	White sugar is less sustainable than brown sugar because a whitening process must be done.
Q2.45	Yogurt made with unpasteurized milk is safe because of a sustainable fermentation process.

Bolt questions are the ones selected by the Cronbach’s alpha and PCA analysis.

**Table 2:** Consumers opinion on food categories issues as affected by the “country” factor for the 19 selected questions.

Question	ANOVA ^†^	Country					
		USA	China	Mexico	Brazil	Spain	India
Bread and cereal products							
Q2.29	NS	4.9	5.1	5.1	5.1	4.9	5.0
Q2.30	***	4.6 c ^‡^	4.9 b	5.2 a	4.9 b	4.8 bc	4.9 b
Sugar and derivatives							
Q2.17	***	4.3 d	5.0 bc	5.3 a	5.1 ab	4.8 c	5.0 bc
Fruits and vegetables							
Q2.26	***	4.8 c	5.1 ab	5.2 a	5.2 ab	4.9 bc	4.5 d
Q2.34	***	5.1 c	5.2 bc	5.8 a	5.1 c	5.4 b	5.4 b
Q2.41	***	4.5 c	4.9 a	5.0 a	4.5 c	4.7 bc	4.8 ab
Fats and oils							
Q2.11	***	4.4 de	4.4 e	5.1 ab	4.9 bc	5.3 a	4.7 cd
Q2.32	***	4.4 d	5.0 a	4.9 ab	4.8 ab	4.5 cd	4.6 bc
Coffee, tea and cocoa							
Q2.4	***	4.4 c	4.7 b	4.8 ab	4.4 c	4.9 a	4.9 a
Q2.18	***	4.5 c	4.7 bc	4.7 bc	5.0 a	4.5 c	4.9 ab
Soft drinks and water							
Q2.21	***	4.9 b	5.6 a	5.6 a	4.8 b	5.8 a	5.0 b
Alcoholic beverages							
Q2.15	***	4.4 c	4.4 c	5.4 a	4.9 b	5.0 b	4.8 b
Meat products							
Q2.2	***	4.0 d	4.3 c	4.8 a	4.7 ab	4.6 bc	3.9 d
Q2.6	***	4.0 bc	3.9 c	4.3 a	4.2 ab	4.0 c	4.3 a
Eggs							
Q2.8	***	4.7 d	5.2 bc	5.1 c	5.4 ab	5.6 a	4.6 d
Q2.23	***	5.2 c	5.6 b	5.9 a	5.9 a	5.9 a	5.2 c
Milk and dairy products							
Q2.25	***	4.0 b	4.7 a	4.0 b	4.0 b	3.8 b	4.8 a
Q2.38	***	4.1 e	4.5 d	5.0 bc	5.4 a	4.9 c	5.3 ab
Q2.40	***	3.8 d	5.0 a	4.4 bc	4.9 a	4.2 c	4.6 b

^†^ NS, not significant (*p* > 0.05) and *** significant differences *p* < 0.001. ^‡^ Values followed by different letters, within the same question, were significantly different (*p* < 0.05).

**Table 3:** Consumers opinion on food categories issues as affected by the “age” and “gender” factors for the 19 selected questions.

Question	ANOVA ^†^	Age				ANOVA ^†^	Gender	
		18–23	24–41	42–52	53–73		Male	Female
Bread and cereal products								
Q2.29	NS	5.0	5.2	5.0	5.0	NS	5.0	5.0
Q2.30	***	5.0 ab^‡^	5.1 a	4.8 b	4.6 c	NS	4.8	4.6
Sugar and derivatives								
Q2.17	NS	4.9	5.0	4.9	4.8	NS	4.8	5.0
Fruits and vegetables								
Q2.26	NS	4.9	5.0	4.9	4.9	NS	4.9	5.0
Q2.34	***	5.2 b	5.5 a	5.3 ab	5.3 b	NS	5.3	5.3
Q2.41	NS	4.7	4.8	4.7	4.6	NS	4.8	4.7
Fats and oils								
Q2.11	***	4.6 b	4.9 a	4.8 a	4.8 a	NS	4.7	4.9
Q2.32	***	4.5 b	4.8 a	4.7 ab	4.7 ab	NS	4.7	4.7
Coffee, tea and cocoa								
Q2.4	***	4.8 a	4.9 a	4.5 b	4.4 b	NS	4.6	4.8
Q2.18	***	4.6 b	4.9 a	4.7 b	4.6 b	NS	4.7	4.7
Soft drinks and water								
Q2.21	***	5.4 a	5.4 a	5.3 ab	5.1 b	NS	5.4	5.2
Alcoholic beverages								
Q2.15	NS	4.7	4.9	4.7	4.9	NS	4.8	4.8
Meat products								
Q2.2	***	4.5 a	4.5 a	4.3 a	4.1 b	NS	4.4	4.4
Q2.6	NS	4.2	4.2	4.0	4.0	NS	4.0	4.2
Eggs								
Q2.8	***	5.1 ab	5.3 a	5.1 a	4.9 b	NS	5.0	5.1
Q2.23	***	5.6 bc	5.8 a	5.7 ab	5.5 c	NS	5.6	5.7
Milk and dairy products								
Q2.25	***	4.3 a	4.4 a	4.1 b	4.0 b	NS	4.3	4.1
Q2.38	***	4.8 ab	5.0 a	4.9 ab	4.7 b	NS	4.8	5.0
Q2.40	NS	4.4	4.6	4.5	4.4	NS	4.5	4.5

^†^ NS, not significant (*p* > 0.05) and *** significant differences *p* < 0.001. ^‡^ Values followed by different letters, within the same question and the same factor, were significantly different (*p* < 0.05). Age: 18–23 years old (Centennials); 24–41 years old (Millennials); 42–52 years old (Gen X) and 53–73 years old (Baby Boomers).

**Table 4:** Consumers opinion on food category issues as affected by the “income” and “education” factors for the 19 selected questions.

Question	ANOVA ^†^	Income (US Dollars)				ANOVA ^†^	Education				
		≤25.000	25.001–50.000	50.001–100.000	>100.000		≤Primary School	High School	Associate’s Degree	Bachelor’s Degree	≥Graduate Degree
Bread and cereal products											
Q2.29	NS	5.0	5.0	5.0	5.2	***	4.4 b^‡^	5.1 a	4.9 a	5.1 a	5.0 a
Q2.30	NS	5.0	4.8	4.8	4.9	***	4.4 b	4.9 a	4.8 a	4.9 a	4.9 a
Sugar and derivatives											
Q2.17	NS	5.0	4.8	4.9	4.9	***	4.6 b	4.7 ab	4.8 ab	5.1 a	5.0 a
Fruits and vegetables											
Q2.26	***	4.9 b	4.9 b	4.9 b	5.2 a	NS	4.9	4.9	4.8	5.0	4.9
Q2.34	NS	5.4	5.3	5.2	5.4	***	4.8 c	5.2 b	5.3 ab	5.4 a	5.4 a
Q2.41	NS	4.7	4.7	4.7	4.8	***	4.2 b	4.6 ab	4.6 ab	4.8 a	4.7 a
Fats and oils											
Q2.11	NS	4.9	4.8	4.7	4.7	NS	4.6	4.8	4.9	4.7	4.9
Q2.32	NS	4.7	4.7	4.6	4.9	***	4.3 b	4.7 a	4.7 a	4.8 a	4.7 a
Coffee, tea and cocoa											
Q2.4	NS	4.7	4.6	4.6	4.8	***	4.5 b	4.5 b	4.5 b	4.8 a	4.7 a
Q2.18	NS	4.7	4.8	4.7	4.9	***	4.4 b	4.7 ab	4.5 b	4.8 a	4.8 a
Soft drinks and water											
Q2.21	NS	5.3	5.3	5.2	5.4	***	4.9 b	5.1 ab	5.4 a	5.4 a	5.3 a
Alcoholic beverages											
Q2.15	***	5.0 a	4.8 b	4.6 b	4.6 b	NS	4.7	4.7	4.8	4.7	4.9
Meat products											
Q2.2	NS	4.4	4.4	4.4	4.5	***	3.9 b	4.2 ab	4.5 a	4.4 a	4.4 a
Q2.6	***	4.2 b	4.1 b	3.9 b	4.7 a	NS	4.3	4.1	4.1	4.1	4.2
Eggs											
Q2.8	NS	5.1	5.1	5.1	5.3	NS	4.8	5.1	5.1	5.2	5.1
Q2.23	NS	5.7	5.6	5.6	5.7	NS	5.6	5.6	5.6	5.6	5.7
Milk and dairy products											
Q2.25	NS	4.2	4.2	4.2	4.3	***	4.0 b	4.2 ab	4.0 b	4.4 a	4.1 ab
Q2.38	***	5.1 a	4.8 b	4.8 b	4.7 b	***	5.1 a	4.8 ab	4.7 b	4.9 ab	5.0 a
Q2.40	***	4.4 b	4.5 b	4.5 b	4.8 a	NS	4.4	4.4	4.4	4.6	4.5

^†^ NS, not significant (*p* > 0.05) and *** significant differences *p* < 0.001. ^‡^ Values followed by different letters, within the same question and the same factor, were significantly different (*p* < 0.05).

**Table 5:** Summary of factors affecting consumers’ attitude towards the sustainability of different food categories. (C1 = Bread and cereal products; C2 = Sugar and derivatives; C3 = Fruits and vegetables; C4 = Fats and oils; C5 = Coffee, tea and cocoa; C6 = Soft drinks and water; C7 = Alcoholic beverages; C8 = Meat products; C9 = Eggs; C10 = Milk and dairy products).

	C1	C2	C3	C4	C5	C6	C7	C8	C9	C10
Country	*^†^	*	***	**	**	*	*	**	**	***
Age	*	NS	*	**	***	*	NS	*	**	**
Gender	NS	NS	NS	NS	NS	NS	NS	NS	NS	NS
Income	NS	NS	*	NS	NS	NS	*	*	NS	**
Education	**	*	**	*	**	*	NS	*	NS	**

^†^ NS, all questions are not significant; *, one question significant different; **, two questions significant different; ***, three questions significant different (*p* > 0.05).

**Table 6:** Mean scores for each consumer cluster on each question used to differentiate among the clusters.

Question	Cluster 1	Cluster 2	Cluster 3
Q2.2	5.02	4.06	1.56
Q2.4	5.41	4.29	1.57
Q2.6	4.64	3.87	1.53
Q2.8	6.00	4.67	1.59
Q2.11	5.46	4.47	1.85
Q2.15	5.33	4.58	1.77
Q2.17	5.83	4.45	1.47
Q2.18	5.64	4.22	1.59
Q2.21	5.71	5.17	2.05
Q2.23	6.39	5.31	1.59
Q2.25	4.67	4.00	1.75
Q2.26	5.54	4.66	1.80
Q2.29	5.83	4.62	1.64
Q2.3	5.69	4.46	1.72
Q2.32	5.45	4.33	1.75
Q2.34	6.05	4.97	2.12
Q2.38	5.76	4.39	1.74
Q2.4	5.35	4.01	1.42
Q2.41	5.37	4.40	1.83
